# Multilineage Differentiating Stress Enduring (Muse) Cells: A New Era of Stem Cell-Based Therapy

**DOI:** 10.3390/cells12131676

**Published:** 2023-06-21

**Authors:** Raghad F. Alanazi, Basma S. Alhwity, Raghad M. Almahlawi, Bashayer D. Alatawi, Shatha A. Albalawi, Raneem A. Albalawi, Amaal A. Albalawi, Mohamed S. Abdel-Maksoud, Nehal Elsherbiny

**Affiliations:** 1 Pharm D Program, Faculty of Pharmacy, University of Tabuk, Tabuk 71491, Saudi Arabia; Raag982@gmail.com (R.F.A.); basma.s19000@gmail.com (B.S.A.); ra.almahallawi@gmail.com (R.M.A.); bashairalatawi@gmail.com (B.D.A.); shatha.zaal@gmail.com (S.A.A.); ranim.ayed0@gmail.com (R.A.A.); amaalalbalawi1421@gmail.com (A.A.A.); 2 Department of Pharmacology & Toxicology, Faculty of Pharmacy, University of Tabuk, Tabuk 71491, Saudi Arabia; mmegahed@ut.edu.sa; 3 Department of Pharmaceutical Chemistry, Faculty of Pharmacy, University of Tabuk, Tabuk 71491, Saudi Arabia; 4 Department of Biochemistry, Faculty of Pharmacy, Mansoura University, Mansoura 35516, Egypt

**Keywords:** muse cells, unique characteristics, applications, clinical trials

## Abstract

Stem cell transplantation has recently demonstrated a significant therapeutic efficacy in various diseases. Multilineage-differentiating stress-enduring (Muse) cells are stress-tolerant endogenous pluripotent stem cells that were first reported in 2010. Muse cells can be found in the peripheral blood, bone marrow and connective tissue of nearly all body organs. Under basal conditions, they constantly move from the bone marrow to peripheral blood to supply various body organs. However, this rate greatly changes even within the same individual based on physical status and the presence of injury or illness. Muse cells can differentiate into all three-germ-layers, producing tissue-compatible cells with few errors, minimal immune rejection and without forming teratomas. They can also endure hostile environments, supporting their survival in damaged/injured tissues. Additionally, Muse cells express receptors for sphingosine-1-phosphate (S1P), which is a protein produced by damaged/injured tissues. Through the S1P–S1PR2 axis, circulating Muse cells can preferentially migrate to damaged sites following transplantation. In addition, Muse cells possess a unique immune privilege system, facilitating their use without the need for long-term immunosuppressant treatment or human leucocyte antigen matching. Moreover, they exhibit anti-inflammatory, anti-apoptotic and tissue-protective effects. These characteristics circumvent all challenges experienced with mesenchymal stem cells and induced pluripotent stem cells and encourage the wide application of Muse cells in clinical practice. Indeed, Muse cells have the potential to break through the limitations of current cell-based therapies, and many clinical trials have been conducted, applying intravenously administered Muse cells in stroke, myocardial infarction, neurological disorders and acute respiratory distress syndrome (ARDS) related to novel coronavirus (SARS-CoV-2) infection. Herein, we aim to highlight the unique biological properties of Muse cells and to elucidate the advantageous difference between Muse cells and other types of stem cells. Finally, we shed light on their current therapeutic applications and the major obstacles to their clinical implementation from laboratory to clinic.

## 1. Introduction

Throughout the past 20 years, significant developments have been achieved in the field of stem cells and regenerative medicine. Stem cells are biological units that are responsible for the regeneration and proliferation of organs and tissue [1]. All types of stem cells share the important characteristic of self-renewal as well as a special capacity for differentiation. Based on their ability to differentiate into specialized cell types, stem cells can be classified into totipotent, pluripotent, multipotent, oligopotent, and unipotent stem cells [2,3]. Each type of stem cells has its advantages. However, pluripotent stem cells (PSCs) either embryonic or induced have been recognized as the gold standard of stem cell-based thereby. This is attributed to their ability to produce a variety of cell types in the body. However, due to risks of immune rejection and teratoma formation, mesenchymal stem cells (MSCs) with multipotent properties have been considered a more suitable alternative [4].

## 2. Multilineage Differentiating Stress-Enduring (Muse) Cells

In 2010, Mari Dezawa and her research group at Tohoku University reported on the isolation of a new subpopulation of MSCs from human bone marrow named Muse cells [5]. The research team indicated that Muse cells could be pluripotent cells that are already present in mesenchymal tissues. These cells were identified as stress-tolerant endogenous pluripotent stem cells that are non-tumorigenic, have self-renewal at a single cell level, and are double positive for (1) the pluripotent marker stage-specific embryonic antigen-3 (SSEA-3) and MSCs marker CD105 if isolated from bone marrow (BM) or organ connective tissue and for (2) SSEA-3 and white blood cell marker CD45 if isolated from peripheral blood [5,6]. The SSEA-3 antibody shows no species differences and can thus be used to characterize Muse cells isolated from different species including human, mouse, rat, rabbit, swine and goat [7]. Muse cells are triploblastic with the ability to differentiate into ectodermal, endodermal and mesodermal lineages. Compared to MSCs, Muse cells demonstrate higher differentiation efficiency and greater integration rate into damaged tissues [8]. Muse cells are different from very small embryonic-like stem cells (VSELs), which are lineage-negative, CD45−, CD34+, CD133+ small sized cells (less than 6 µm length) located mainly in BM [9].

Regarding tissue distribution, Muse cells have unique and distinctive distribution in the living organism compared with other somatic stem cells. They are widely residing in the connective tissue of every organ. Muse stem cells have been successfully isolated from BM-MSCs, dermal fibroblastic tissue [5], and adipose tissue. In BM, Muse cells reside in the BM cavity rather than connective tissue, comprising about 0.03% of the mononucleated cell fraction. They are also present in the peripheral blood at the range of 0.01~0.2% [8,10,11,12]. Additionally, they can be found in extraembryonic tissues, i.e., the umbilical cord. Unlike other somatic cells, Muse cells do not have their own niche in connective tissue. However, they are found freely and sparsely distributed, which may be explained by their endogenous active dynamic movement [7].

It is presumed that under basal conditions, Muse stem cells are mobilized from BM to various body organs through peripheral blood. This rate of mobilization varies per individual. Additionally, this rate changes within the same individual based on physical conditions and the existence of injury/disease [8]. Along this vein, clinical studies reported increased circulating Muse cells in patients with ischemic stroke and myocardial infarction during acute phases of diseases [13,14].

## 3. Characteristics of Muse Stem Cells

**Figure 1:**
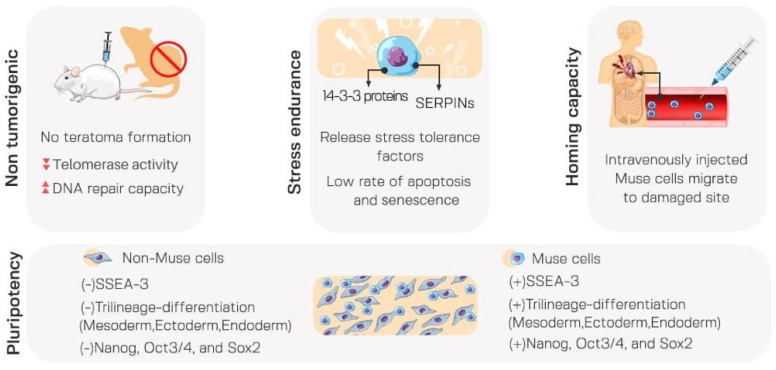
Distinctive characteristics of Muse cells compared to other types of stem cells. Muse cells are characterized by non-tumorigenicity, stress tolerance, high homing capacity to injured site and pluripotency.

Muse cells have unique and distinctive characteristics summarized in Figure 1.

### 3.1. Pluripotency

Pluripotent stem cells are self-renewable cells and can generate all types of cells in the endodermal, mesodermal, and ectodermal lineages. Interestingly, Muse cells qualify as pluripotent stem cells, as they have both abilities at a single cell level. Based on pluripotency, MSCs can be segregated into Muse and non-Muse cells. Non-Muse cells do not survive or proliferate in single-cell suspension. As a result, they cannot form pluripotent-like clusters. Concurrently, pluripotency markers such as sex-determining region Y box 2 (Sox2), octamer binding transcription factor ¾ (Oct3/4), reduced expression 1 (Rex1) and Nanog are either undetectable or significantly lower in non-Muse cells compared to Muse cells [8]. The lower rate of MSCs differentiation after integration into damaged tissues could be attributed to the low percentage of Muse cells (1%) that can be found in the MSCs population [8]. Intriguingly, the in vitro rate of differentiation of Muse cells can be up-regulated (~80–95%) in the presence of a certain set of cytokines.

Despite the self-renewability and triploblastic differentiation exhibited by Muse cells, the source from which they were isolated defines their differentiation directivity. For instance, compared to BM and dermal-derived Muse cells, Muse cells derived from adipose tissue show a higher tendency to differentiate into adipocytes, skeletal muscle cells and osteocytes. BM-derived Muse cells show a higher differentiation tendency into pancreatic and hepatic cells. Dermal fibroblast and BM-derived Muse cells possess higher levels of genes that direct their differentiation into melanocyte, neuronal, and epidermal cells. Muse cells derived from peripheral blood are unique in expressing CD45 as well as higher levels of pluripotency genes compared to Muse cells from other sources [15].

### 3.2. Stress Tolerance

Muse cells were first identified as a stress-endurance subpopulation of MSCs that tolerate about 16 h of incubation in trypsin without nutrition [16]. Muse cells also show a highly conserved cellular mechanism that is essential for cell survival and proliferation in response to extreme cellular stress [17]. They secrete stress-related factors including 14-3-3 proteins and serpins when they are active. Serpins belong to a family of proteins with the protease inhibition effect and are only expressed in Muse cells and not MSCs cells. The 14-3-3 proteins are 30 kDa highly conserved acidic molecules that defend against stress-induced apoptosis and help damaged proteins keep their physiologically important structure [4,18]. Hence, Muse cells demonstrate lower rates of senescence and apoptosis compared to non-Muse MSCs. Additionally, Muse cells can quickly and effectively sense DNA damage and activate the DNA damage repair system, thus increasing their resistance to genotoxic stressors compared to MSCs and non-Muse cells [6].

### 3.3. Non-Tumorigenicity

The fundamental challenge in determining the cell population most suitable for cell- based regenerative therapy is tumorigenicity [19]. Muse cells are non-tumorigenic cells, proliferating at a rate of 1.3 days/cell division in both adherent and suspended states. Tumorigenic factors vary across embryonic/induced pluripotent stem cells (ES/iPS) and Muse cells. In general, those factors are substantially expressed in ES/iPS cells, whereas Muse cells have very low expression. It is noteworthy that telomerase activity, a sign of tumorigenic activity, is noticeably low in Muse cells when compared to iPS and Hela cells [20]. In fact, no teratoma formation was seen for up to 6 months after transplanting human BM- and adipose-derived Muse cells into the tissues of immunodeficient mice [12]. The higher DNA repair capacity of Muse cells increases resistance to mutation and lowers the risk of tumorigenesis [7].

Lin28 and Let7 maintain balance between tumorigenesis and pluripotency. The overexpression of Let-7, an upstream modulator of genes involved in cell cycle division, blocks Lin28 gene expression. In contrast, Lin28 expression, an oncogene, degrades Let-7. A high Lin28/Let7 ratio was observed in embryonic and induced PSCs (iPSCs), which is likely responsible for their tumorigenesis characteristics. However, Let-7 overexpression was observed in Muse cells and is assumed to be responsible for suppressing Lin28 expression, preventing tumorigenesis and promoting tissue regeneration [17].

Another interesting difference between Muse cells and embryonic and iPSCs is that Muse stem cells rely on the fibroblast growth factor family to maintain their self-renewal and proliferation abilities. On the other hand, embryonic and iPSCs depend on Leukemia Inhibitory Factor (LIF) and bone morphogenic protein 4 (BMP4) for their proliferation. All these facts highlight the distinctive nature of Muse cells with pluripotent ability accompanied by low incidence of tumorigenesis, making them favorable to embryonic and iPSCs that are associated with high tumorigenesis risk [10].

### 3.4. High Homing Capacity of Muse Cells (Selective Homing to Injured Tissues)

**Figure 2:**
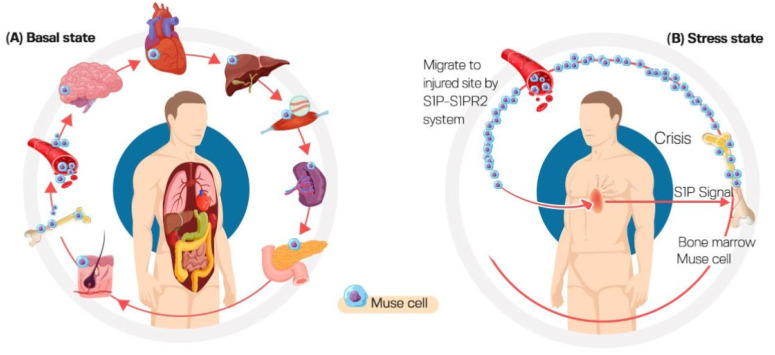
Tissue distribution of Muse cells in (**A**) steady state and in (**B**) stress state. Under normal conditions, Muse cells constantly move from bone marrow to peripheral blood to supply various body organs. Under stress conditions, Muse cells migrate to the injured site through the sphingosine-1-phosphate (S1P)–S1PR2 axis.

Muse cells are generally dormant, only becoming active in the presence of extremely high levels of cellular stress [5]. Both BM-derived and intravenous administered Muse cells can identify the location of the injured site and migrate specifically to the damaged site. Sphingosine-1-phosphate (S1P), the alerting signal, tends to attract Muse cells to the site of damage primarily through the sphingosine-1-phosphate receptor 2 (S1PR) expressed on the Muse cells’ surface, permitting them to preferentially home to the site of injury [21,22], as shown in Figure 2.

Sato et al. reported that Muse cells comprise 0.04% ± 0.003% of the mononuclear cells in human peripheral blood. They are positive for SSEA-3 and CD45 with 10.1 ± 0.3 µm size. About 94% of the Muse cells population in peripheral blood was positive for major histocompatibility complex class II molecule HLA-DR. This contrasts with BM-Muse cells which were reported to be negative for HLA-DR. In addition, about 85% of peripheral blood Muse cells were positive for B cell and follicular dendritic cells marker CD19. They expressed the pluripotency markers Oct3/4, Nanog, and Sox2 at significantly higher levels compared to BM-Muse cells. They also expressed sphingosine-1-phosphate (S1P) receptor 2 and migrated toward S1P. These cells also showed low adherence and low proliferation in vitro, preventing their adherence to vessel walls and subsequent emboli formation [23].

The importance of the S1P–S1PR2 axis for Muse cells’ reparative activity was emphasized by the study of Yamada et al. [24] in which rabbit Muse cells but not non-Muse cells migrated toward an S1PR2-specific agonist SID46371153 in a dose-dependent manner. This activity was suppressed by S1PR2 gene silencing in Muse cells. In contrast, the migration of Muse cells, but not non-Muse cells, toward acute myocardial infarction heart was inhibited in the presence of S1PR2 antagonist JTE-013 in a dose-dependent manner. The co-injection of JTE-013 reduced the integration of Muse cells into post-infarct heart in vivo, resulting in larger infarct size compared to allografts without JTE-013.

According to clinical evidence from patients with acute myocardial infarction and stroke, plasma S1P levels rise before endogenous peripheral blood–Muse cells activation, demonstrating the capacity of Muse cells to detect damage signals, autonomously homing to injured locations when delivered intravenously [13,24].

### 3.5. Adherent–Suspension Switch

Unlike MSCs which survive mainly in an adherent system and hematopoietic cells which survive basically in a suspension system, Muse cells can survive in both suspension and adherent culture. Interestingly, the adherent–suspension switch regulates their pluripotency. When Muse cells are transferred to suspension, the expression of pluripotency markers Sox2, Nanog and Oct3/4 increases fifty to several hundred times compared to adherent culture. However, levels of pluripotent markers return to original levels upon changing to the adherent culture [25]. Moreover, these markers translocate into the nucleus, where they act as transcription factors, when the cells are transferred to a suspension [10]. Additionally, adherent–suspension switch changes the epigenetics of Muse cells. A lower methylation of Sox2, Nanog and Oct3/4 promoter regions has been reported in suspension-cultured Muse cells compared to adherent-cultured Muse cells. Endogenously, at the basal level, Muse cells reside in an adherent state with low pluripotency capacity. This can be attributed to the existence of pluripotency genes in the cytoplasm with an inability to exert their function. Under stress, Muse cells detach from tissue and circulate in blood in suspension status, thus increasing their pluripotency capacity [26].

### 3.6. Anti-Immunity

Muse cells are anti-immune. They express high levels of the immune modulatory enzyme indoleamine 2,3-dioxygenase (IDO) and human leukocyte antigen G (HLA-G), which are known to suppress both humoral and cellular immunities. Indeed, the HLA-G mechanism is responsible for avoiding the immune rejection of placenta and fetus in mammals [27]. Interestingly, when entering infarcted rabbit hearts, 87.5% of the Muse cells have been reported to express HLA-G, which is a much higher rate compared to the 20% reported by MSCs. Moreover, intravenously administrated Muse cells can survive as differentiated cells in host tissue for more than 6 months even without the need for immunosuppressive treatment [24]. Such an anti-immune characteristic makes Muse cells useful not only in tissue repair but also for suppressing autoimmunity [28].

## 4. Isolation of Muse Cells

**Figure 3:**
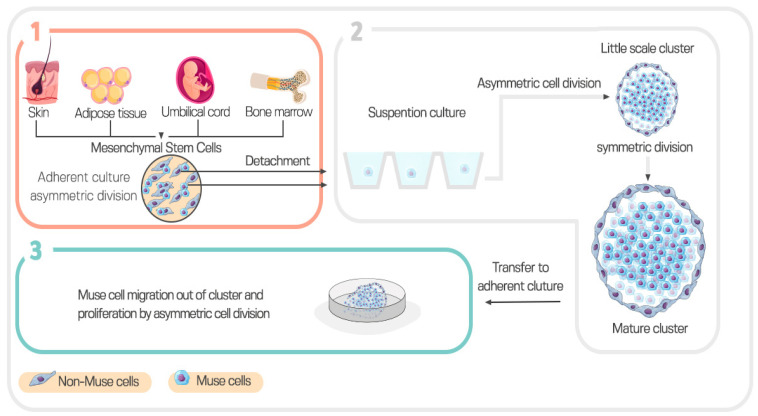
Steps for separation and expansion of Muse cells. (**1**) Adherent culture, (**2**) Suspension culture, (**3**) Adherent culture.

Muse cells cannot be distinguished from MSCs in adherent culture. However, they form pluripotent cell clusters upon transfer to suspension culture. Muse cells can be isolated from cultured BM, dermal fibroblasts, or adipose tissue-derived MSCs that are either commercially available or by primary culturing of tissue source [28]. Muse cells can then be obtained from cultured MSCs by magnetic-activated cell sorting (MACS) or fluorescence-activated cell sorting (FACS). A Muse cell proliferates in an asymmetric manner in an adherent culture state, generating one new Muse cell in addition to one non-Muse cell. However, in the suspension culture state, Muse cells initially proliferate by asymmetric division, producing several non-Muse cells. The flat slender non-Muse cells surround Muse cells, forming an ensheathment. Entrapped Muse cells then proliferate by symmetric division, generating a mature cluster of 50–150 μm size within two weeks. When a mature cluster is cultured in adherent status, the inner Muse cells proliferate by asymmetric division after migration out of the cluster [4]. Steps for the isolation and expansion of muse cells are summarized in Figure 3.

## 5. Intravenous Administration of Muse Cells

**Figure 4:**
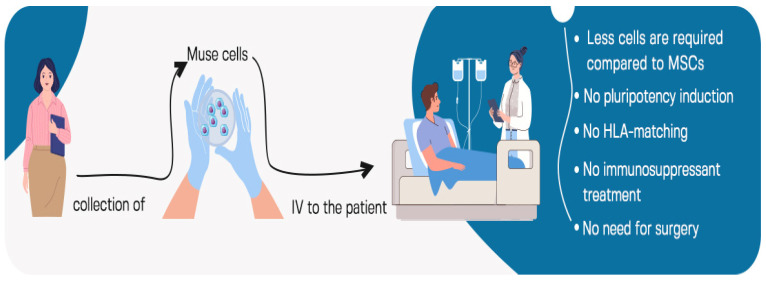
The concept of intravenous administration of Muse cells. Three steps are required for Muse cells infusion: collection from the donor, expansion and then intravenous administration.

Due to the higher ability of Muse cells to target damaged tissues, fewer cells are required for intravenous administration compared to MSCs. CL2020 is a clinical-grade Muse cell-based product manufactured by Life Science Institute, Inc. (Tokyo, Japan) that contains 1.5 × 10^7^ cells per 15 mL of frozen preparation [29]. Muse cells are administrated by intravenous infusion with no need for surgery. Importantly, Muse cells possess immunomodulatory characteristics. Thus, their administration does not require immunosuppressant treatment or HLA matching. In addition, no cytokine treatment or gene introduction are required to induce Muse cell pluripotency and/or trigger their differentiation. Once infused, Muse cells exert their trophic, anti-inflammatory and anti-apoptotic activities in host tissue for extended period. All these facts allow for the medical implementation of Muse cells in clinics and general hospitals in addition to specialized medical institutions. Indeed, only three steps are required for Muse cells infusion in clinical trials: collection from the donor, expansion and then intravenous administration [7], as shown in Figure 4.

## 6. Therapeutic Applications of Muse Cells

Muse cells have been investigated in a wide range of disorders including acute myocardial infarction (MI), cerebrovascular disease, chronic kidney disease, and liver damage. The number of Muse cells in the peripheral blood of MI patient has been reported to be positively correlated with functional recovery [30]. Along this line, the administration of exogenous naive Muse cells devoid of cytokine or gene modification has been proposed to strengthen reparative activity after damage brought on by an ischemic event. Muse cells have been seen to heal a variety of tissues, including the dermis, liver, kidney, heart, and brain [31]. In the following sections, we review preclinical studies applying Muse cells in various diseases.

### 6.1. Stroke

Stroke represents the second leading cause of death worldwide [32]. It is a cerebrovascular disorder that can be either ischemic or hemorrhagic and is caused by arterial blockage or the abrupt rupture of cerebral blood vessels. Hemorrhagic stroke is classified as subarachnoid hemorrhage and intracerebral hemorrhage. Ischemic stroke is provoked by an embolism, atherothrombosis, or small vessel disease, which causes a low level of cerebral blood flow. Ischemic stroke is the prevalent form representing 80% of stroke cases. The current treatment strategies for stroke include mechanical thrombectomy and the intravenous injection of recombinant tissue plasminogen activator. However, due to the limited treatment time frame, contraindications, and risk of complications, these revascularization therapies are only appropriate for less than 10% of patients [33]. Stroke-induced neuronal damage along with the lack of neuronal regenerative ability account for disability and mortality. Therefore, it is essential to develop a novel therapy to encourage functional recovery and re-establish the damaged neural circuit [18]. In vitro, Muse cells can be spontaneously differentiated or induced into neural stem cells or neural lineage cells [15,34]. Table 1 summarizes the preclinical studies of Muse cells on stroke.

### 6.2. Myocardial Infarction

Acute myocardial infarction (AMI) is one of the major causes of morbidity and death worldwide. It is caused by coronary artery occlusion due to plaque rupture [30]. Due to left ventricular (LV) dysfunction and remodeling, AMI frequently causes severe heart damage that results in heart failure. The current treatment strategy applies percutaneous coronary intervention to re-perfuse occluded coronary artery. To be effective, reperfusion should be achieved within 60 min after AMI onset. However, the prognosis is worse if it exceeds 120 min [39]. BM MSCs, BM-mononucleated cells and iPSCs have been reported to repair damaged myocardium [40]. Indeed, various preclinical studies showed their capability of differentiating into vascular smooth muscle cells, endothelial cells, and even cardiac myocytes. Additionally, MSCs have been reported to produce paracrine growth factors that enhance angiogenesis and promote the survival of nearby cardiomyocytes [41,42]. However, clinical application of the aforementioned cells is still limited. Compared to MSCs, Muse cells demonstrated a high rate of engraftment (about 14%) to the infarct area when administrated intravenously [30]. Tanaka et al. reported a positive correlation between the number of Muse cells in the peripheral blood of AMI patients and good prognosis in terms of improved remodeling and LV function. However, the authors concluded that endogenous Muse cells could not be enough for full reparative efficacy. Hence, boosting their efficacy could be achieved by the exogenous administration and/or development of strategies that enhance their mobilization during the onset of surgery [14]. Table 2 summarizes preclinical studies applying Muse cells in MI.

### 6.3. Neuronal Diseases

#### 6.3.1. Amyotrophic Lateral Sclerosis (ALS)

Amyotrophic lateral sclerosis (ALS) is a neurodegenerative disorder affecting upper and lower motor neurons leading to stiffness, muscle weakness, and hyperreflexia due to motor neurons degeneration [44]. Muse cells intravenously administrated to a mouse model of ALS homed to the spinal cord and differentiated into glial fibrillary acidic protein-positive astrocytes. Treatment resulted in improved behavioral tests, enhanced the survival of motor neurons, suppressed neuronal atrophy, and increased lower limb muscle strength [45].

#### 6.3.2. Perinatal Hypoxic Ischemic Encephalopathy

Perinatal hypoxic ischemic encephalopathy occurs due to hypoxic–ischemic events during the prenatal period, preventing adequate blood supply to the infant’s brain and leading to long-term devastating neurologic disorders such as mental retardation, severe seizure, and/or cerebral palsy. In an experimental model of perinatal hypoxic–ischemic encephalopathy induced in 7-day-old rats, intravenously administrated Muse cells improved motor and cognitive functions, suppressed microglial activation and modulated glutamate metabolism [46].

### 6.4. Diabetes Mellitus

Approximately half a billion individuals worldwide are affected by diabetes mellitus (DM), which is an endocrine-metabolic illness that is extremely common and is growing at an alarming rate. It is characterized by hyperglycemia attributed to either a lack of insulin (Type I DM), peripheral insulin resistance and/or insufficient insulin synthesis (Type I DM). Type 1 diabetes is characterized by an autoimmune-mediated destruction of beta cells of the pancreas [47]. Antigen-presenting cells (APCs) in Type I DM patients detect autoantigens generated by damaged cells (independent of the underlying stimulation) and trigger pro-inflammatory responses. β cell damage is triggered by pro-inflammatory cytokines produced by immune cells and is accelerated by oxidative stress status. Type II DM develops because of insulin resistance, which makes cells incapable of handling high blood glucose levels and ultimately causes them to die by apoptosis. The involvement of the immune system has also been reported. Therefore, Muse cells may be beneficial in DM by either protecting pancreatic β cells from death or modulating the action of the uncontrolled immune system [48]. Furthermore, Kinoshita et al. have reported a beneficial effect of Muse cells in healing of skin ulcers generated in type 1 diabetes immunodeficient mice. In this study, Muse cells-treated ulcers showed faster healing with thick epidermis [49].

### 6.5. Spinal Cord Injury

Spinal cord injuries (SCIs) are considered as a major contributor for impairment. In addition, significant neurological or psychological deficits are a side effect of SCI that undoubtedly adds to the overall burden of this disorder. They lead to a shorter life expectancy, a loss of productive years, and high health care expenses [50]. A cascade of damaging processes, including oxidative stress, inflammatory events, ischemia, and neurological defects are all part of the pathogenesis of SCI [51].

Mesenchymal stem cells (MSCs) and neural precursor cells (NPCs) both are promising strategies to cure SCI. However, their use is limited. The ability of NPCs to differentiate into neurons and glial cells made a promising strategy for treating SCI, but they are not used due to ethical concerns. MSCs on the other hand, play a crucial role in motor function recovery, the inhibition of apoptosis, and the protection of neurons. The low survival rate of MSCs after transplantation in SCI is the main cause of not using MSC in treating SCI. In addition to the low survival rates MSCs also have a low ability to differentiate neuron lineages [52].

Interestingly, recent in vitro and in vivo studies induced human Muse cells into neural precursor cells (Muse-NPCs) to determine if Muse-NPCs can function as seed cells for the repair of SCI by examining its ability of differentiation and in vivo performance. Muse-NPCs have the features of neural stem cells since they can differentiate into oligodendrocytes, neurons, and astrocytes. Most significantly, Muse-NPCs had a greater differentiation rate and more noticeable benefits in biological activities when compared to BMSCs and Muse cells [52] (Table 3).

### 6.6. Damaged Intestinal Epithelial Cells of Rat

One of the important factors in the pathophysiology of inflammatory bowel disease (IBD) is the immune response. A variety of inflammatory markers are involved, most notably tumor necrosis factor-α (TNF-α) that was found to be associated with the intestinal damage that accompanies the disease. In this context, Sun et al. [54] demonstrated that Muse cells separated from BM-MSCs had anti-inflammatory and immunomodulatory effects on damaged intestinal epithelial cells in vitro, suggesting great potential for Muse cells in the treatment of inflammatory bowel disease and other intestinal inflammatory disorders.

### 6.7. Acute Lung Ischemia–Reperfusion Injury in a Rat Model

Lung ischemia–reperfusion injury (IRI) has a great impact on the failure of lung transplantation and lung malfunction thereafter, affecting both graft function and patients’ survival. This is mainly because of the dysregulation of blood flow to the organ with the subsequent stimulation of various immune responses. Indeed, several inflammatory agents can eventually damage the structural integrity of the graft, including cytokines, neutrophils and ROS. Compared to MSCs, Muse cells demonstrated superior efficacy in terms of homing to injured lung, histological injury score on hematoxylin–eosin sections, alveolar–arterial oxygen gradient, arterial oxygen partial pressure to fractional inspired oxygen ratio and left lung compliance. These optimistic findings encourage further investigation toward the applicability of Muse cells in treating and preventing transplant lung IRI [55].

### 6.8. Bladder Inflammation

Interstitial cystitis (IC) is a chronic inflammatory bladder condition that affects females more than males [56]. Its pathophysiology is divided into two categories: non-Hunner type IC and Hunner type IC with Hunner lesions. Hunner’s lesions are only found in 4–10% of individuals who have either recently been diagnosed with interstitial cystitis or bladder pain syndrome (IC/BPS) or who have IC/BPS as a suspect. There are many theories explaining the etiology of bladder inflammation. Regardless, the precise cause of the illness is unknown; one possible etiology is related to mast cells. It is thought that when mast cells become activated and infiltrate the bladder wall, they may release mediators that cause an inflammatory reaction in the bladder tissues, increasing epithelial permeability. This allows the urine components to leak through the bladder wall and leads to hypersensitivity of the nerves. This inflammatory mechanism is likely to be engaged in fibrogenesis and reduce bladder capacity through mastocytosis, which is particularly evident in people with Hunner type IC (HIC) [57]. Locally injected Muse cells into the anterior and posterior bladders of a HCl-induced Hunner-type interstitial cystitis-like rat model resulted in improved nociceptive behavior, attenuated inflammation, and overactivity, suggesting Muse cells as promising treatment strategy for HIC [58].

### 6.9. Pancreatitis

Acute pancreatitis is an inflammatory condition that affects the exocrine pancreas and is accompanied by tissue damage and necrosis. Acute pancreatitis can vary in intensity; it can be moderate or severe; it may resolve on its own; or it can lead to extra pancreatic organ failure, systemic inflammatory response syndrome, and even death. Unfortunately, it is significant to note that there is no current effective therapy that can change the course of the disease. Supportive treatment and fluid resuscitation are the current management [59]. Muse cells’ intravenous administration in a mouse model of severe acute pancreatitis induced by taurocholate reduces edema, macrophage infiltration, and apoptosis and increased the proliferation of pancreatic cells [60].

### 6.10. Aortic Aneurism

Aortic aneurysms (AAs) are life-threatening enlargements of the aorta caused by multi-stage tissue degradation that results from oxidative stress, inflammation, and atherosclerosis. Lymphocytes and monocytes/macrophages infiltrate the aortic wall and activate matrix metalloproteinases as well as inflammatory cytokines and chemokines, resulting in the loss of structural support provided by endothelial cells (ECs), vascular smooth muscle cells (VSMCs), and the extracellular matrix (ECM). Therefore, this eventually results in aneurysmal dilatation that is permanent and poses a gradual risk of rupture as the diameter grows over time [61].

Treatments for AA include prosthetic graft replacement and endovascular repair, which is considered to prevent an anticipated rupture. However, there is a need for less invasive treatment, since open surgery is frequently associated with mortality and morbidity. As part of AA therapy, earlier research attempted to reduce inflammation. However, the findings are insufficient to justify clinical advice [62]. Interestingly, the intravenous administration of human bone marrow-derived Muse cells into an experimental model of AA induced by the periaortic incubation of CaCl_2_ and elastase in immunodeficient mice reduced aneurysm size and improved its dilation. When injected, Muse cells were found to migrate to the aneurysm and spontaneously dedifferentiated into VSMCs and ECs while retaining elastic fibers [63].

### 6.11. Hepatectomy

Various types of liver cancers are most effectively treated with hepatectomy. However, one of the fatal consequences of this procedure is post-hepatectomy liver failure (PHLF), which usually occurs when the removed volume of the liver exceeds 75% of the total volume. In an attempt to save the liver in urgent instances, liver transplantation is recommended. Yet, the morality of using a liver graft to treat PHLF is still up for question, and little is known about how well liver transplantation works in this condition. Hence, the necessity for a novel therapeutic approach for PHLF is critical [64]. The administration of Muse cells collected from swine bone marrow-mesenchymal stem cells (MSCs) through the portal vein of a swine model of 70% hepatectomy suppressed inflammatory and fibrotic reactions and improved liver function, as demonstrated by reduced bilirubin level and improved blood clotting. This was accompanied by efficient incorporation into the liver and differentiation into hepatocyte marker-positive cells, and trophic effects. In addition, no evidence of portal vein hypertension or thrombosis was reported, which is common for cell infusion via the portal vein. Thus, the safety and efficacy of Muse cells in providing the functional recovery and reparative effect in an experimental model of hepatectomy suggests their promising use as a therapeutic strategy for PHLF [65].

### 6.12. Chronic Kidney Disease

Renal dysfunction is caused by many diseases and conditions. Severe and persistent renal dysfunction may lead to end-stage renal disease (ESRD). Except for maintenance dialysis or kidney transplantation, there are no available therapeutic modalities for ESRD. Thus, regenerative medicine represents promising renal therapeutics [66]. Focal segmental glomerulosclerosis is characterized by extensive glomerular scarring, which is a common precursor to chronic kidney disease [19]. Systemically administrated Muse cells into a mouse model of focal segmental glomerulosclerosis migrated into injured glomeruli, and it also spontaneously differentiated into various glomerular cells including endothelial cells, podocytes and mesangial cells, leading subsequently to the attenuation of glomerular sclerosis and restoration of renal functions [67].

## 7. Clinical Trials Using Muse Cells

Based on promising findings of the preclinical studies, many clinical trials have tested Muse cells for the treatment of stroke, myocardial infarction, spinal cord injury, neonatal cerebral palsy, dystrophic epidermolysis bullosa and ALS [7]. In a clinical trial for myocardial infarction (JapicCTI-183834 and JapicCTI-195067), the intravenous administration of CL2020 (Life Science Institute, Inc., Tokyo, Japan) demonstrated safety, improvement of left ventricular ejection fraction and wall motion score index [29]. To investigate dystrophic epidermolysis bullosa, a non-randomized, single-arm, non-controlled clinical trial (JapicCTI-184563) in patients with more than 4 weeks recurrent ulcers and refractory ulcers was selected. Interestingly, CL2020 infusion improved skin erosion area and reduced the ulcer size [68]. Another single-center, open-label, dose-escalation clinical trial using CL2020 is currently in phase I, assessing the safety and the tolerability of CL2020 cells in hypoxic ischemic encephalopathy in neonates receiving hypothermia therapy (ClinicalTrials.gov Identifier: (NCT04261335), jRCT2043190112). CL2020 cells were also used in a randomized, double-blind, placebo-controlled clinical trial for cerebral infarction. CL2020 administration for 52 weeks attenuated the severity of neurological disorders and improved the motor function of both upper and lower extremities (JapicCTI-184103). Additionally, another exploratory clinical trial held by the Life Sciences Institute in Japan is currently testing CL2020 cells for the treatment of amyotrophic lateral sclerosis (jRCT2063200047) as well as spinal cord injury (JapicCTI-194841). Furthermore, the Life Sciences Institute in Japan initiated a clinical trial using CL2020 for the treatment of acute respiratory distress syndrome related to SARS-CoV-2 (jRCT2043210005).

## 8. Challenges in Therapeutic Application of Muse Cells

Muse cells’ expansion to an adequate number for clinical administration takes time, and the culture cost is relatively expensive. Additionally, complex steps of isolation and proliferation may result in inconsistent quality between different batches of cell products. Hence, innovative time-saving and cost-effective isolation and enrichment methods for Muse cells are in great demand [18].

## 9. Conclusions and Future Perspectives

Muse cells represent a more realistic choice for next-generation cell therapy. These unique cells herald a brave new world of tissue protection and repair. Golden standardization is urgently needed to produce high-quality and high-consistency Muse cells. Deeper research will make this cell source a clinically competitive treatment method in the near future.

## Figures and Tables

**Table 1:** Preclinical studies using Muse cells in stroke.

Model	Stem Cells Source	Results	Reference
Middle cerebral artery occlusion in immunodeficient mice	Human BM MSCs-derived Muse cells	Muse cells were integrated into the peri-infarct cortex, spontaneously differentiated into neuronal markers (Tuj 1 and NeuN)-positive cells, replaced lost neurons and restored motor function	[35]
Transient middle cerebral artery occlusion in rats	Human fibroblast-derived Muse cells	Muse cells integrated with brain microenvironment demonstrated a high rate of differentiation into neuronal cells with the subsequent reconstruction of the neuronal circuit and alleviation of stroke symptoms	[34]
Subacute lacunar stroke model in immunodeficient mice	Human BM MSCs-derived Muse cells	Muse cells differentiated into neural cells, facilitated neural recovery, improved behavioral score, and demonstrated solid safety outcomes over the experimental period	[36]
Immunodeficient mouse lacunar stroke model	Clinical-grade multilineage-differentiating stress-enduring cell-based product CL2020	CL2020 was safe with no tumorigenesis or adverse effects detected. CL2020 migrated to the peri-infarct area, expressed neuronal markers, and showed functional recovery	[37]
Mouse intracerebral hemorrhage (ICH) model	Human BM MSCs-derived Muse cells	Muse cells resided in the ICH brain, differentiated into NeuN and MAP-2 positive neurons and improved survival rate and motor function	[38]

**Table 2:** Preclinical studies using Muse cells in MI.

Model	Stem Cells Source	Results	Reference
Swine model of acute myocardial infarction	Semi-clinical grade human Muse cell product	Muse cells homed into the infarct border area, differentiated into cardiomyocytes (Troponin I positive) and microvessels (CD31-positive) reduced infarct size, improved the left ventricular (LV) function and remodeling	[43]
Rabbit acute myocardial infarction model	Human BM-MSCs derived Muse cells	Muse cell xenografts and allografts successfully engrafted, reduced infarct size and restored functions. Allografts resided in the tissue and maintained functional recovery for up to 6 months with no need for immunosuppressive treatment	[24]

**Table 3:** Preclinical studies applying Muse cells in SCI.

Model	Stem Cells Source	Results	Reference
Rat model of thoracic spinal cord contusion injury	Clinical product CL2020 containing 300,000 Muse cells	Muse cells in CL2020 differentiated into neuronal cells with improvement of hindlimb motor function, smaller cystic cavity and preservation of 5-hydroxytryptamine (5-HT) fibers	[53]
Spinal cord injury induced in rats	BM-MSCs-derived muse cells induced into neural precursor cells	Restoration of motor function	[52]

## Data Availability

The data presented in this study are available in the article.
